# Correlation between serum lipid levels and endocrine resistance in patients with ER-positive breast cancer

**DOI:** 10.1097/MD.0000000000035048

**Published:** 2023-10-13

**Authors:** Hong Sun, Congting Hu, Xiaohan Zheng, Jie Zhuang, Xiaoxia Wei, Jiaqin Cai

**Affiliations:** a Department of Pharmacy, Shengli Clinical Medical College of Fujian Medical University, Fujian Provincial Hospital, Fuzhou, P. R. China; b School of Pharmacy, Fujian Medical University, Fuzhou, P. R. China; c Department of Pharmacy, South Branch of Fujian Provincial Hospital, Shengli Clinical Medical College of Fujian Medical University, Fuzhou, P. R. China.

**Keywords:** breast cancer, clinical stage, endocrine resistance, estrogen receptor-positive, LDL-C, serum lipid, TG

## Abstract

Lipid metabolism may be involved in the development of endocrine drug resistance in ER-positive (ER+) breast cancer (BC). This study aimed to investigate the relationship between serum lipid levels, risk stratification of dyslipidemia, and endocrine resistance. We collected the data from 166 ER + breast cancer patients who received endocrine therapy (ET). 73 of 166 patients (44.0%)developed endocrine resistance. Univariate and multivariate COX regression were conducted to explore the potential factors affecting endocrine resistance in BC. The clinical T stage, mean serum lipid levels in ET progression-free-survival (total cholesterol, triglycerides, low-density lipoprotein cholesterol, apolipoprotein A, and triglycerides/high-density lipoprotein cholesterol) were correlated with endocrine resistance (*R* = 0.214, *P* = .006; *R* = 0.268, *P* < .001; *R* = 0.182, *P* = .019;*R* = 0.197, *P* = .011; *R* = 0.211, *P* = .006; *R* = 0.159, *P* < .041). Clinical stage, triglycerides (TG) in endocrine therapy progression-free-survival (ePFS) and low-density lipoprotein cholesterol (LDL-C) in ePFS were independent predictors of endocrine resistance (*P* < .05; OR = 1.406, CI 1.108–1.783, *P* < .05; OR = 1.309, CI 1.026–1.669, *P* < .05, respectively). Moreover, in clinical stage III, the ePFS was worse in patients with in the high-risk and extremely high-risk group the median ePFS time was 8.0 months (95% CI: 1.140–14.860, *P* < .05). Clinical stage, TG in ePFS and LDL-C in ePFS may act as a new predictive biomarker for endocrine resistance in BC. The lipid levels of BC patients should be closely monitored throughout the treatment process, and patients with dyslipidemia should receive treatment immediately.

## 1. Introduction

In the world, breast cancer (BC) is the most common cancer and the leading cause of death among women.^[1]^ ER-positive (ER+) breast cancer has the highest incidence rate and approximately 80% of all cases are present.^[2]^ Endocrine therapy (ET) is one of the standard treatments for estrogen receptor-positive BC and is mainly divided into selective estrogen receptor modulators and aromatase inhibitors.^[3]^ While this form of breast cancer is often successfully targeted with endocrine therapies against ER + receptors, roughly 20% of cases are initially resistant to ET, with another 30 to 40% of cases developing resistance over time.^[4]^

The mechanism of endocrine resistance is still under discussion, and it can be linked to lipid reprogramming in BC.^[5,6]^ Lipid metabolic reprogramming is a significant characteristic of numerous malignant tumors.^[7]^ This process encompasses several aspects, including the dynamic equilibrium between the augmentation of de novo fatty acid synthesis and fatty acid oxidation, alterations in the lipid composition of biofilm to facilitate proliferation, and modifications in lipid signaling molecules that promote tumor progression.^[8]^ Furthermore, the up-regulation of lipid metabolism-related proteins in tumors can stimulate the emergence of malignant phenotypes.^[9]^ Previous studies have reported the correlation between lipids and breast cancer prognosis.^[10]^ Total cholesterol is an independent predictor of breast cancer risk.^[11]^ Good control of serum lipid metabolism may improve the tumor immune microenvironment and prognosis in postmenopausal Hormone receptor-positive/HER2-negative breast cancer patients.^[12]^ The cholesterol metabolite 27-hydroxycholesterol was found to induce the proliferation of estrogen receptor-positive breast cancer cells and facilitate metastasis.^[13,14]^ In BC endocrine therapy, Tamoxifen reduced total cholesterol (TC) and low-density lipoprotein cholesterol (LDL-C) levels.^[15]^ Tamoxifen binds with high affinity to the microsomal antiestrogen binding site and inhibits cholesterol esterification at therapeutic doses.^[16]^ There have also been some reports of lipid metabolism involved in ET, lipid metabolism is involved in tamoxifen resistance and cholesterol metabolism plays a role in tamoxifen resistance.^[5,17]^ Basic research on the relation between lipid reprogramming and endocrine resistance in BC is currently being studied by many researchers. But there no relevant clinical study has reported the relation of lipid reprogramming and endocrine resistance in BC. Most of these previous studies focus on the serum lipid level at a certain cutoff time, while ignoring the impact of the full-course lipid level on the prognosis of endocrine drug resistance in breast cancer.

Hence, our study aims to explore the relationship between lipid levels and endocrine resistance in ER + breast cancer patients and to provide a basis for clinical judgment on whether early intervention of lipid levels is needed to reduce the occurrence of endocrine resistance and improve the quality of life of patients.

## 2. Materials & methods

### 2.1. Study population

In this retrospective study, we recruited 166 women who received ET at Fujian Provincial Hospital with histologically proven ER + breast cancer from January 2014 to July 2022. These patients met the following inclusion criteria: regular follow-up without loss of follow-up or death; invasive ductal carcinoma of the breast confirmed by pathology, estrogen receptor-positive in immunohistochemistry; patients receiving regular adjuvant ET; head, chest, and abdomen computed tomography, magnetic resonance imaging, whole body bone scan, or whole body PET-CT is one of the tests to determine the disease No distant metastasis occurred; and regular reexamination and fasting blood lipid test in the morning, with relatively complete biochemical test results. The exclusion criteria were: patients with poor treatment compliance; patients with a history of other malignant tumors; and patients without standard treatment. The TNM staging method was referred to by the American Joint Committee on Cancer 8th edition TNM staging system for breast cancer. The study is approved by the Institutional Research Ethics Committee of Fujian Provincial Hospital (approval number # K2019-01-047).

### 2.2. Information collection

The data on the clinicopathological features were collected, which included the age, height, weight, menstrual status, T stage, N stage, M stage, Ki67, imaging data, the baseline serum lipid level, and the serum lipid levels in ET progression-free-survival. Among these, the cutoff value for Ki-67 is 30%, with greater than or equal to the cutoff value indicating strong expression and less than the cutoff value indicating low expression.^[18]^ Endocrine therapy progression-free-survival (ePFS) is defined as the time from the start of ET to the onset of endocrine resistance, and ePFS is measured in months. Mean serum lipid levels in ePFS are defined as the mean of serum lipids from the onset of endocrine medication until the onset of endocrine resistance, and the serum lipid index is averaged 5 or more times per sample. The automatic biochemical analyzer in our hospital was used to measure the concentration of TC, triglyceride (TG), LDL-C, high-density lipoprotein cholesterol (HDL-C), apolipoprotein A (Apo A), apolipoprotein B (Apo B), and Apo B/Apo A in the serum of the patients. The criteria for determining hypercholesterolemia were referred from those prescribed by the Joint Committee for the Development of Chinese Adult Dyslipidemia Prevention and Control Guidelines,^[19]^ as follows: TC > 6.1 mmol/L, LDL-C > 3.12 mmol/L any indicator exceeds the upper limit; lipid ratio AI = (TC-HDL-C)/HDLC, THR = TG/HDL-C, LHR = LDL-C/HDL-C. According to Chinese expert consensus on lipid management in patients with malignancy (2021 edition),^[20]^ considering the influence of 10-year risk factors of Atherosclerotic Cardiovascular Disease events, patients undergoing early treatment will continue to be divided into low-risk, medium-risk, high-risk, and extremely high-risk. Considering that the interventions of low-risk and medium-risk were the same, and the interventions of high-risk and extremely high-risk were consistent, we divided them into 2 groups according to the intervention: low-risk and medium-risk, high-risk and extremely high-risk.

### 2.3. Outcome

The criteria for recurrence and metastasis were based on new response evaluation criteria in solid tumors: revised RECIST guideline (version 1.1).^[21]^ According to the 5th ESO-ESMO international consensus guidelines for advanced breast cancer (ABC 5),^[22]^ endocrine resistance is described as either primary or secondary endocrine resistant breast cancer. Primary endocrine resistance is defined as relapse while on the first 2 years of adjuvant ET, or disease progression within the first 6 months of first-line ET for advanced breast cancer (ABC), while on ET. Secondary endocrine resistance is defined as relapse while on adjuvant ET but after the first 2 years, or relapse within 12 months of completing adjuvant ET, or disease progression ≥6 months after initiating ET for ABC, while on ET. According to the definition, 73 of 166 patients (44.0%) developed endocrine resistance.

### 2.4. Statistical analytic tools and methods

SPSS 25.0 was used for statistical analyses. Firstly, continuous variables were presented as mean ± standard deviation or median with interquartile range, as appropriate. Categorical variables were presented as proportions. The 2-tailed t-test or Mann–Whitney *U* test was conducted for all continuous variables and the χ^2^-test or Fisher exact test for categorical variables, as appropriate, to identify candidate covariates. The relationship between endocrine resistance and the factors with a *P* value <.05 in univariate analysis were analyzed by the Spearman correlation test, the point-biserial correlation, the Mantel-Haenszel chi-squared test. Then, the independent risk factors influencing prognosis were analyzed with the occurrence of endocrine resistance as the dependent variable and the factors with a *P* value <.15 in the univariate analysis as the independent variable by multivariate COX regression. Factors with *P <* .05 in the COX regression analysis were considered independent factors. The survival curve showed that the risk grade of dyslipidemia affected the drug-resistant survival of ER+ breast cancer patients under different cancer stages. (*P* < .05 was considered a significant difference).

## 3. Results

### 3.1. The relationship between clinicopathological characteristics and endocrine resistance

A total of 166 patients with ER + breast cancer without distant metastasis were enrolled in this study. Among them, 73 patients (44.0%) developed endocrine resistance. The comparison of clinicopathological characteristics between the 2 groups showed that with the increase of T stage, the patients were more prone to endocrine resistance ((*R* = 0.214, *P* < .05, Tables 1 and 3). Endocrine resistance was not associated with age, BMI, menopausal status, clinical N stage, clinical stage, and Ki67 (Table 1).

**Table 1:** Demographic and clinical characteristics of patients according to endocrine resistance status.

Characteristics	Expression of the experimental groups		
	Nonresistant (n = 93, 56.0%)	Resistant (n = 73, 44.0%)	*P*
Age (yr)	50 ± 13	47 ± 10	.111
BMI (kg/m^2^)			.547
BMI < 24	64 (68.8)	47 (64.4)	
BMI ≥ 24	29 (31.2)	26 (35.6)	
Menopausal status			.572
Premenopausal	52 (55.9)	44 (60.3)	
Postmenopausal	41 (44.1)	29 (39.7)	
Clinical T stage			.006
T1	37 (39.8)	21 (28.8)	
T2	52 (55.9)	37 (50.7)	
T3	3 (3.2)	12 (16.9)	
T4	1 (1.1)	3 (4.1)	
Clinical N stage			.053
N0	51 (54.8)	34 (46.6)	
N1	29 (31.2)	16 (21.9)	
N2	4 (4.3)	12 (16.4)	
N3	9 (9.7)	11 (15.1)	
Clinical stage			.052
I	25 (26.9)	18 (24.7)	
II	55 (59.1)	31 (42.5)	
III	13 (14.0)	24 (32.9)	
Ki67			.211
Ki67 < 30%	34 (36.6)	20 (27.4)	
Ki67 ≥ 30%	59 (63.4)	53 (72.6)	

Data are given as mean ± standard deviation if calculated using the independent samples t-test, and as n (%) if calculated using the chi-square test and as median (interquartile range) if calculated using the Mann–Whitney *U* test.

BMI = body mass index, BMI = weight (kg)/height*height (m^2^), N = nodal, T = tumor.

### 3.2. The relationship between serum lipid and endocrine resistance

There were no significant differences in baseline serum lipids between the nonresistant group and resistant groups. Endocrine resistance is also the lack of association with risk stratification of dyslipidemia and hypercholesteremia. But in the comparison of mean serum lipid levels in ePFS, TG, TC, LDL, Apo A, and THR levels are positively associated with endocrine resistance (*R* = 0.268, *P* < .001; *R* = 0.182, *P* < .05; *R* = 0.197, *P* < .05; *R* = 0.211, *P* < .05; *R* = 0.159, *P* < .05). Details are shown in Tables 2 and 3.

**Table 2:** Relationships between serum lipid and endocrine resistance.

Characteristics	Expression of the experimental groups		
	Nonresistant (n = 93, 56.0%)	Resistant (n = 73, 44.0%)	*P*
Baseline serum lipid levels			
TG	1.06 (0.80, 1.40)	1.22 (0.89, 1.67)	.093
TC	4.87 ± 1.12	4.94 ± 1.01	.706
HDL-C	1.28 (1.05, 1.56)	1.24 (1.08, 1.54)	.786
LDL-C	3.23 ± 1.01	3.29 ± 0.90	.665
Apo A	1.35 (1.17, 1.49)	1.30 (1.20, 1.52)	.875
Apo B	0.95 (0.79, 1.16)	0.92 (0.78, 1.10)	.549
Apo B/Apo A	0.69 (0.59, 0.88)	0.71 (0.58, 0.84)	.734
AI	2.47 (2.09, 3.68)	2.77 (2.08, 3.43)	.952
THR	0.78 (0.53, 1.26)	0.93 (0.62, 1.44)	.276
LHR	2.31 (1.86, 3.25)	2.50 (1.86, 3.11)	.781
Mean serum lipid levels in ePFS			
TG	1.30 (1.00, 1.76)	1.63 (1.27, 2.36)	.001
TC	4.69 (4.04, 5.42)	5.01 (4.60, 5.66)	.019
HDL-C	1.23 ± 0.32	1.28 ± 0.29	.303
LDL-C	3.11 ± 0.88	3.46 ± 0.88	.011
Apo A	1.29 (1.17, 1.48)	1.43 (1.26, 1.55)	.007
Apo B	0.96 (0.80, 1.19)	0.99 (0.82, 1.14)	.479
Apo B/Apo A	0.71 (0.58, 0.95)	0.69 (0.57, 0.89)	.456
AI	2.83 (2.16, 3.17)	3.07 (2.14, 3.99)	.430
THR	1.06 (0.70, 1.68)	1.29 (0.88, 2.09)	.042
LHR	2.63 (1.88, 3.29)	2.73 (1.95, 3.50)	.331
Risk stratification of dyslipidemia			.49
Low-risk and medium-risk	78 (83.6)	64 (87.7)	
High-risk and extremely high-risk	15 (16.1)	9 (12.3)	
Hypercholesteremia	41 (44.1)	31 (42.5)	.834
Statins	21 (22.6)	13 (17.8)	.37
No	74 (76.4)	62 (84.9)	
Yes	19 (20.4)	11 (15.1)	

Data are given as mean ± standard deviation if calculated using the independent samples t-test, and as n (%) if calculated using the chi-square test and as median (interquartile range) if calculated using the Mann–Whitney *U* test.

Apo A = apolipoprotein A, mmol/L, Apo B = apolipoprotein B, mmol/L, Apo B/Apo A = apolipoprotein B/ apolipoprotein A, Baseline-AI = (TC-HDLC)/ HDLC, HDL-C = high density lipoprotein cholesterol, mmol/L, LDL-C = low density lipoprotein cholesterol, mmol/L, LHR = LDL-C/ HDL-C, Mean serum lipid levels in ePFS = the mean of serum lipids from the onset of endocrine medication until the onset of endocrine resistance, and the serum lipid index was averaged 5 or more times per sample, ePFS is measured in months, TC = total cholesterol, mmol/L, TG = triglycerides, mmol/L, THR = TG/ HDL-C.

**Table 3:** Correlations between potential influencing factors and endocrine resistance.

Factor	Resistant	
	r	*P*
Clinical T stage	0.214	.006
Mean serum lipid levels in ePFS		
TG	0.268*	<.001
TC	0.182	.019
LDL-C	0.197	.011
Apo A	0.211	.006
THR	0.159	.041

Mean serum lipid levels in ePFS, the mean of serum lipids from the onset of endocrine medication until the onset of endocrine resistance,and the serum lipid index was averaged 5 or more times per sample, and ePFS is measured in months.

Apo A = apolipoproteinA, mmol/L, LDL-C = low density lipoprotein cholesterol, mmol/L, TC = total cholestero, mmol/Ll, TG = triglycerides, mmol/L, THR = TG/high density lipoprotein cholesterol.

* Correlation is significant at the 0.01 level (2-tailed). The correlation analysis between Clinical T stage and resistant (yes or no) is conducted using the Mantel-Haenszel chi-squared test. Since LDL-C is a normally distributed continuous variable, the correlation analysis between LDL-C and resistant (yes or no) is conducted using the point-biserial correlation. TG, TC, Apo A, and THR are non-normally distributed continuous variables, so the correlation analysis between them and resistant (yes or no) is conducted using the Spearman correlation test.

### 3.3. COX regression to identify the value of clinical stage and mean serum lipid levels in the ePFS (TG, LDL) in predicting the effect

The independent risk factors influencing prognosis were analyzed with the occurrence of endocrine resistance as the dependent variable and the factors with a *P* value <.15 in the univariate analysis as the independent variable by multivariate COX regression. Clinical T stage, clinical N stage, clinical stage, baseline serum lipid levels (TG) and mean serum lipid levels in ePFS (TG, TC, LDL-C, Apo A, THR) were candidate predictors of therapeutic effect. The COX regression analysis suggested that the clinical stage and mean serum lipid levels in ePFS (TG, LDL) could serve as the independent predictor of therapeutic effect (*P <* .05). Details are shown in Table 4.

**Table 4:** Independent predictors of endocrine resistance by multivariate analysis.

	Coefficient	OR (95% CI)	*P*
Clinical stage	/	/	.001
I*II	−0.110	0.896 (0.496–1.617)	.714
I*III	0.936	2.549 (1.367–4.752)	.003
ePFS-TG	0.340	1.406 (1.108–1.783)	.005
ePFS-LDL-C	0.269	1.309 (1.026–1.669)	.030

Independent predictors of endocrine resistance in the multivariate analysis were analyzed by COX regression.

ePFS-LDL-C = low density lipoprotein cholesterol in endocrine therapy progression-free-survival, mmol/L, ePFS-TG = triglycerides in endocrine therapy progression-free-survival, mmol/L.

### 3.4. Effect of dyslipidemia risk stratification on ePFS in ER-positive breast cancer patients

Patients were assigned to 2 groups according to the dyslipidemia risk assessment criteria: low-risk and medium-risk groups (n = 142), high-risk and extremely high-risk groups (n = 24). The proportion of deletion in low-risk and medium-risk groups, high-risk and extremely high-risk groups were 54.9% and 62.5%, respectively. See Supplemental Digital Content (Table S1), http://links.lww.com/MD/J877.

**Figure 1.**
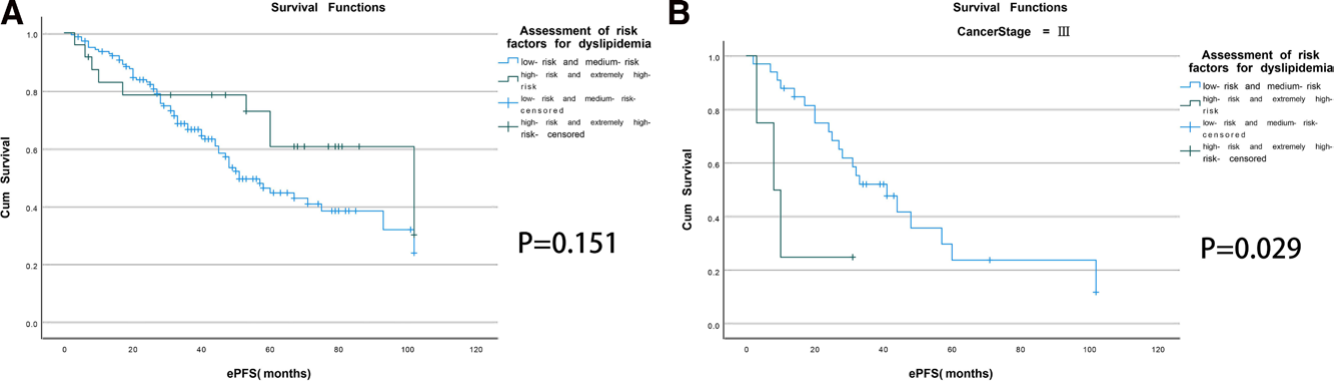
Cumulative disease-free survival curves. Kaplan–Meier plots according to endocrine therapy progression-free-survival and risk stratification of dyslipidemia (A), endocrine therapy progression-free-survival and risk stratification of dyslipidemia in clinical stage III (B).

The Kaplan–Meier survival curve showed that the risk stratification of dyslipidemia had no significant effect on the ePFS (Fig. 1A). To further clarify the specific lipid index affecting ePFS in breast cancer, we explored the effect of risk stratification of dyslipidemia on endocrine resistance in breast cancer under different clinical stages. The Kaplan–Meier survival curve showed the ePFS was worse in patients with in the high-risk and extremely high-risk groups than the median ePFS time was 8.0 months in clinical stage III (95% CI: 1.140–14.860) (Fig. 1B).

## 4. Discussion

ET is the primary treatment modality in patients with ER + breast cancer. However, a large proportion of patients develop drug resistance during ET, which is also the main cause of disease recurrence and metastasis, and ultimately death.^[4]^ The mechanism of endocrine resistance is still under discussion, and it may be related to lipid reprogramming in breast cancer.^[5]^

Previous clinical studies have reported that dyslipidemia is a high risk factor for the poor prognosis of BC.^[10,23]^ And activation of cholesterol and fatty acid metabolism is considered as drivers of endocrine resistance.^[24]^ However, in BC endocrine therapy, Tamoxifen reduced TC and LDL-C levels.^[15]^ Tamoxifen binds to microsomal antiestrogen binding sites with high affinity and inhibits cholesterol esterification at therapeutic doses.^[16]^ On the basis that ET is beneficial to controlling serum lipid levels, but lipid-related components are the factors driving endocrine resistance, we wanted to explore whether serum lipids have good predictive indicators for early screening of endocrine resistance. Therefore, we collected clinical pathology data of patients receiving ET and conducted a series of analyses on the relationship between endocrine resistance and clinical pathology characteristics, the relationship between serum lipid indicators and endocrine resistance, the influence of dyslipidemia risk grade on endocrine resistance, and the relationship between blood lipid interventions and endocrine resistance.

Previous clinical studies have reported that dyslipidemia is a high-risk factor for poor prognosis in BC,^[10]^ and also reported that baseline dyslipidemia is a good prognostic factor for breast cancer.^[10,25]^ It is unclear whether there is a correlation between the above 2 findings. In conclusion, current studies on the role of serum lipids in breast cancer prognosis are still controversial. Our results showed no significant association between baseline serum lipid levels and endocrine resistance in breast cancer patients, but a significant association between mean serum lipid levels in ePFS and endocrine resistance. Our study further divided the serum lipid levels into baseline serum lipid levels and mean serum lipid levels in ePFS. Unlike previous research on breast cancer prognosis, there may be differences in the results with baseline serum lipid levels, possibly due to the unstable nature of lipid levels at a single time point. Mean serum lipid levels in ePFS can better represent the overall lipid levels during the entire treatment period compared to a single time point. Additionally, we found a significant correlation between average blood lipid levels during ET resistance and endocrine resistance, providing further support for fatty acid metabolism is drivers of endocrine resistance.^[24]^

Subsequently, according to Chinese expert consensus on lipid management in patients with malignancy (2021 edition),^[20]^ considering the influence of ASCVD event 1 0 years of risk factors and intervention measures, patients continue to be divided into low-risk and medium-risk groups and high-risk and extremely high-risk group, to explore the correlation with the ePFS of the patient, the results showed no significant difference (Fig. 1A). Because clinical stage is also an independent risk factor for developing endocrine drug resistance. Therefore, we further explored whether there was a correlation between risk stratification of dyslipidemia and ePFS at different clinical stages, and the results showed that the ePFS was worse in patients with in the high-risk and extremely high-risk group that the median ePFS time was 8.0 months at clinical stage III (95% CI: 1.140–14.860, *P* < .05).

Some studies have reported that statins can improve prognosis in breast cancer,^[26,27]^ but the mechanisms by which statins improve cancer prognosis are unclear. We collected data on statin samples (including irregular doses), the study results did not show an association with endocrine resistance, and the lack of statistical difference here may be due to the small sample size.

The strength of this study is that we collected and analyzed the whole-process blood lipid data. However, this study has some limitations, such as the retrospective study design. The short follow-up period may affect the results of the prognosis analysis. In addition, our data were collected from a single center.

## 5. Conclusions

According to this study, clinical stage, TG in ePFS and LDL-C in ePFS act as new predictive biomarkers for endocrine resistance in breast cancer. The lipid levels of breast cancer patients should be closely monitored throughout the treatment process, and patients with dyslipidemia should receive treatment immediately. To demonstrate these possible mechanisms, more research is needed.

## Acknowledgments

We would like to thank the patients who participated in this study.

## Author contributions

**Conceptualization:** Hong Sun.

**Data curation:** Congting Hu.

**Formal analysis:** Congting Hu, Xiaohan Zheng.

**Funding acquisition:** Hong Sun, Jiaqin Cai, Jie Zhuang, Xiaoxia Wei.

**Investigation:** Hong Sun, Congting Hu.

**Methodology:** Hong Sun.

**Writing – original draft:** Congting Hu, Xiaohan Zheng.

**Writing – review & editing:** Jie Zhuang, Xiaoxia Wei, Jiaqin Cai.

## Supplementary Material
