# Supplementary material for: Correlation between serum lipid levels and endocrine resistance in patients with ER-positive breast cancer
Source: Medicine (Baltimore). 2023 Oct 13;102(41):e35048. doi: 10.1097/MD.0000000000035048 (PMC10578763; doi:10.1097/MD.0000000000035048)
Supplement: Supplementary file 1 [file medi-102-e35048-s001.docx]

**Table S1** **Management of endocrine resistance cases**

| Assessment of risk factors for dyslipidemia | Total N | resistant | non-resistant | |
| --- | --- | --- | --- | --- |
|  |  |  | N | Percent |
| low-risk and medium-risk | 142 | 64 | 78 | 54.9% |
| high-risk and extremely high-risk | 24 | 9 | 15 | 62.5% |
| Overall | 166 | 73 | 93 | 56.0% |

**Abbreviations:N,The number of cases of ER-positive breast cancer.**
